# Chondroitin sulfate in invertebrate development

**DOI:** 10.1002/pgr2.70009

**Published:** 2024-11-05

**Authors:** Ayano Moriya, Eriko Nakato, Jin-Ping Li, Hiroshi Nakato

**Affiliations:** 1 Department of Genetics, Cell Biology, and Development, University of Minnesota, Minneapolis, Minnesota, USA; 2 Department of Medical Biochemistry and Microbiology, Uppsala University, Uppsala, Sweden

**Keywords:** basement membrane, *Caenorhabditis elegans*, Chondroitin sulfate, development, *Drosophila*

## Abstract

Chondroitin sulfate (CS) is one of the most evolutionarily conserved glycosaminoglycans (GAGs). Although CS’s function in skeletal development is well established in vertebrates, CS exists in more primitive animal species with no cartilage or bone, such as *C. elegans* and *Drosophila*, indicating that the original role of CS was not in the skeletal system. In this review, we focus on the roles of CS and the mechanisms of action during development of two genetically trackable model organisms, *C. elegans* and *Drosophila*.

## *C. elegans* CS

### *C. elegans* CS in vulval development and embryonic cytokinesis

Chondroitin sulfate (CS) is an evolutionarily conserved class of glycosaminoglycans. The backbone of CS is composed of repeating disaccharide units of glucuronic acid (GlcA) and *N*-acetylgalactosamine (GalNAc). The assembly of CS chains is initiated by the synthesis of the linkage tetra-saccharide, GlcAβ1–3Galβ1–3Galβ1–4Xylβ1-O-, which is covalently attached to specific serine residues of a core protein.

CS research in the nematode *Caenorhabditis elegans* (*C. elegans*) has a rich history, which started about 25 years ago with both biochemical and genetic studies. On the biochemical side, structural analyses revealed that *C. elegans* contains a large amount of chondroitin (Table 1).^1,7,9^ Unlike vertebrates and *Drosophila*, this glycosaminoglycan appeared to be unsulfated in the nematode. Because of this, it has been historically called chondroitin. As we discuss below, however, a small amount of sulfation was detected later.^10,11^ Therefore, in this review, we will use the terms of CS/CSPGs for this *C. elegans* glycosaminoglycan and its proteoglycans instead of chondroitin/CPGs.

**FIGURE 1:**
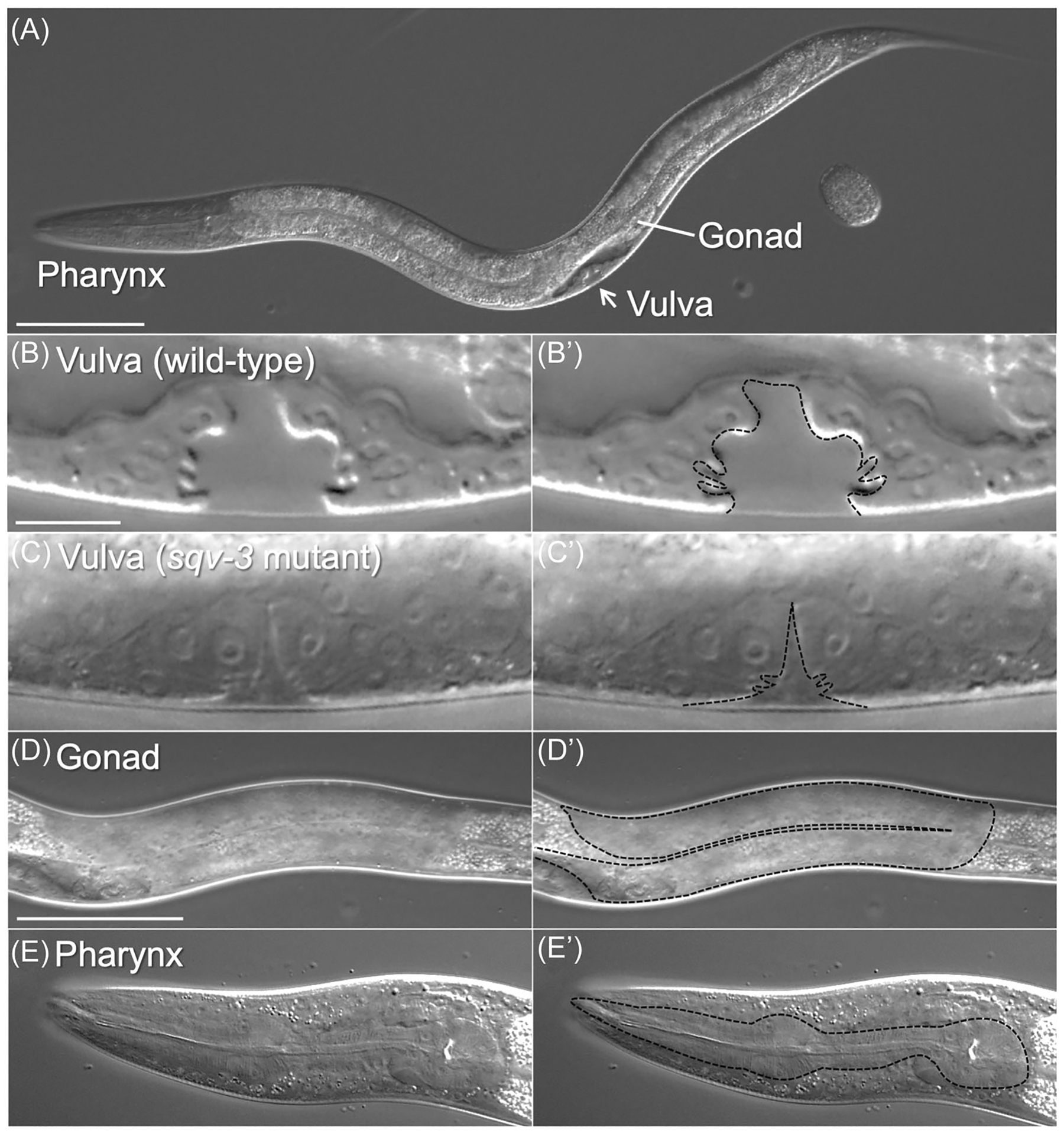
*C. elegans* vulva, gonad, and pharynx. (A) Wild-type L4-stage *C. elegans*. (B-E’) Vulva from wild-type (B and B’) and *sqv*-*3(n2842)* mutant (C and C’), wild-type gonad (D and D’), and wild-type pharynx (E and E’) are shown. Dotted lines indicate each structure (B’, C’, D’, and E’). Scale bars: 100 μm (A), 10 μm (B-C’), and 50 μm (D-E’).

Independent of these biochemical analyses, a genetic study was conducted to elucidate the molecular mechanisms of epithelial invagination during development. The invagination of epithelial cell layers, which can lead to the formation of tubular structures, is a fundamental process of morphogenesis and repeatedly occurs during embryogenesis. A powerful forward genetic study by Herman and Horvitz used the morphogenesis of *C. elegans* vulva as a model system^12,13^ (Figure 1). The vulva develops from ventral epidermal precursors where a set of specialized epithelial cells are specified as vulval precursors.^14^ A single cell of the somatic gonad, called the anchor cell, breaks the basement membrane (BM) and forms a hole in the epidermis. Subsequently, the vulval cells invaginate and create a tube that connects the hermaphrodite uterus to the external environment. In wild-type hermaphrodites, the vulval cells invaginate by the mid-L4 stage (Figure 1). An elegant genetic screen was designed to identify mutant animals in which the space between the invaginating vulval cells and the cuticle was absent or collapsed, showing the failure of the invagination and/or tube formation (Figure 1). Importantly, they focused on mutations that perturb vulval invagination without affecting vulval cell lineage, which means that the isolated mutants have the normal number of vulval cells. The genetic screen yielded 25 mutations, comprising 8 complementation groups, which were named *squashed vulva* (*sqv*) 1–8. Detailed analyses using electron microscopy showed that in addition to the severely reduced vulval extracellular space, the mutant vulval area was found to be more electron dense than that of the wild type, suggesting a change in the extracellular environment. Due to the failure of normal egg laying, *sqv* hermaphrodites tend to be bloated with unlaid eggs.

**FIGURE 2:**
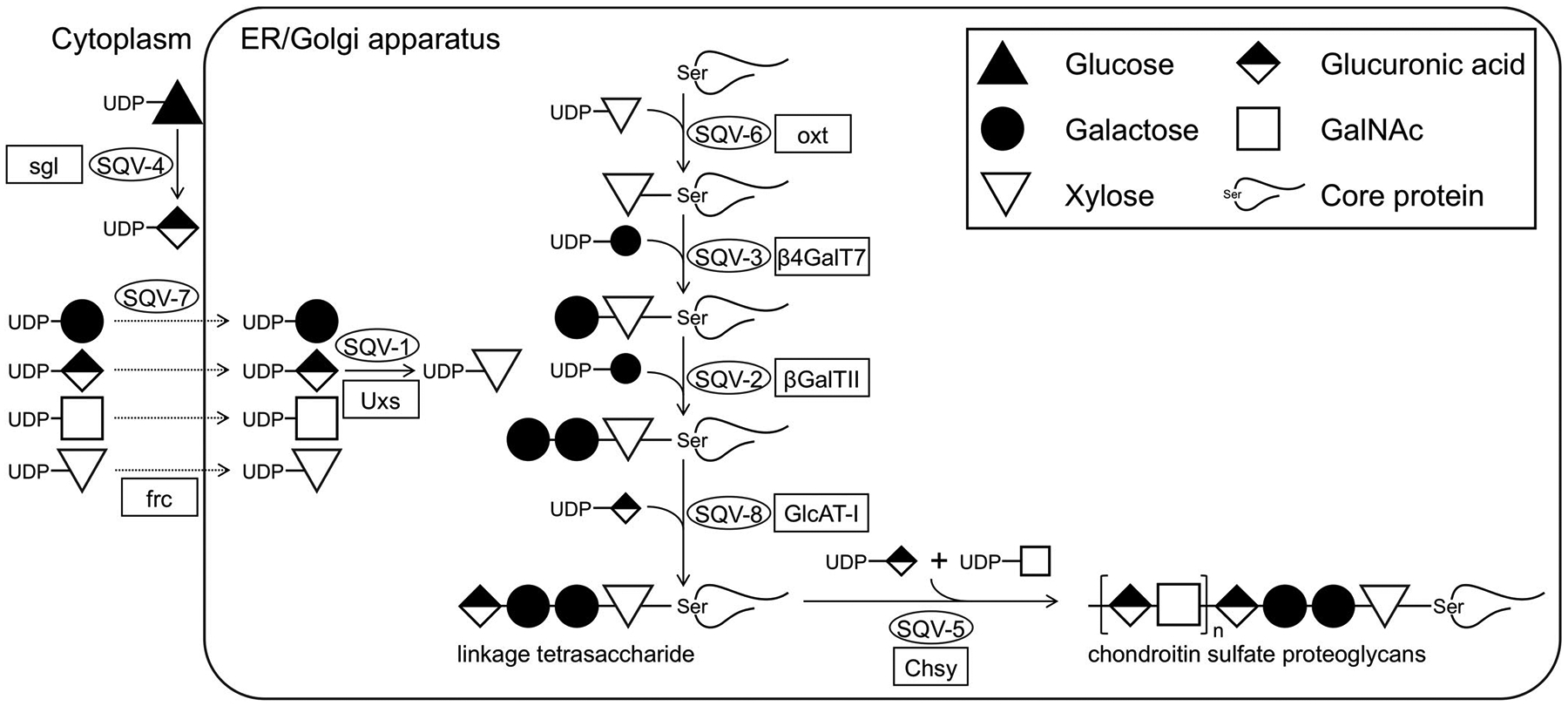
*C. elegans sqv* genes in CS biosynthesis. SQV-4 converts UDP-glucose (UDP-Glu) to UDP-glucuronic acid (UDP-GlcA). SQV-7 transports UDP-GlcA, UDP-galactose (UDP-Gal), and UDP-*N*-acetylgalactosamine (UDP-GalNAc) from the cytoplasm to lumen of the Golgi apparatus. SQV-1 converts UDP-GlcA to UDP-xylose (UDP-Xyl). CS is covalently attached to specific Serine residues in core proteins through common GAG-protein linker region tetrasaccharide, GlcAβ1–3Galβ1–3Galβ1–4Xylβ1-O-. This structure is formed by a series of reactions catalyzed by four SQV enzymes: SQV-6 (xylosyltransferase), SQV-3 (galactosyltransferase I), SQV-2 (galactosyltransferase II), and SQV-8 (glucuronosyltransferase I). SQV-5 (*N*-acetylgalactosaminyltransferase-I and II) functions in CS chain initiation and elongation. *Drosophila* orthologues of *sqv* genes are shown with rectangles. Modified from Hwang et al.^19^

Remarkably, we later learned that all eight genes isolated in this screen function in one biological process: CS biosynthesis. Molecular cloning of the first seven out of eight genes suggested that these molecules were commonly required for the biosynthesis of both heparan sulfate (HS) and CS^15–19^ (Figure 2). They were predicted to function in the syntheses of UDP-GlcA (SQV-4) and UDP-Xyl (SQV-1), the transport of the substrates to the Golgi (SQV-7), and the formation of the linkage tetra-saccharide for HS and CS (SQV-6, 3, 2, and 8). Biochemical experiments supported these conclusions for each gene.

The identity of the last *sqv* gene, *sqv*-*5*, was revealed by two studies.^20,21^ Hwang et al. demonstrated that *sqv*-*5* encodes a protein similar to human chondroitin synthase (ChSy) and chondroitin *N*-acetylgalactosaminyltransferase (ChGn) (Figure 2). Biochemical evidence confirmed that SQV-5 is responsible for the biosynthesis of CS but not HS. Mizoguchi et al. took a reverse genetics approach and landed on the same gene.^21^ A *C. elegans* homolog of ChSy was cloned, and RNAi knockdown animals and isolated mutants exhibited embryonic lethality and a cell division defect (discussed later) that are consistent with *sqv* mutants.

Interestingly, SQV-5 was found to be a bifunctional glycosyltransferase. CS chain initiation requires *N*-acetylgalactosaminyltransferase-I (GalNAcT-I), while chain elongation needs *N*-acetylgalactosaminyltransferase-II (GalNAcT-II) and glucuronosyltransferase-II (GlcAT-II) activities. In humans, chain elongation is catalyzed by the action of the complex of chondroitin synthase (ChSy) and chondroitin promoting factor (ChPF).^22^ The chain is initiated by chondroitin *N*-acetylgalactosaminyltransferase (ChGn): ChSy lacks this activity. Interestingly, however, SQV-5 is the only protein significantly similar to the human ChSy/ChGn in the *C. elegans* genome. In vitro enzymatic assays demonstrated that SQV-5 indeed possesses both GalNAcT-I and -II activities,^20,21^ suggesting it is responsible for both CS chain initiation and elongation. *C. elegans* does have the ChPF homolog, encoded by *mig*-*22*.

The finding of SQV-5 being the *C. elegans* ChSy/ChGn demonstrated that depletion of CS, not HS, causes *sqv* phenotypes. What is the function of CS during vulva development? Although we do not know the exact mechanism by which CS regulates vulva invagination and tube formation, there are examples for CS’s functions in similar morphogenetic processes. For example, in the sea urchin embryo, CS-containing matrix is secreted onto the apical surface of a specific region of an epithelial sheet.^23^ It was proposed that the hydration of this newly deposited matrix drives the folding of the epithelium. Therefore, *C. elegans* CS may promote the expansion of the invagination space or shape vulval lumen by forming a gel-like scaffold that swells with water to exert outward pushing forces.^24^ Alternatively, CS may provide the vulval epithelium ECM with the rigidity required to generate the invagination space.

### *C. elegans* CS in cytokinesis and distal tip cell migration

The role of CS in nematode development is not limited to the vulval invagination and tube formation- *sqv* mutations disrupt other developmental processes. One striking phenotype of *sqv* mutants is the failure of cytokinesis during the first cell division of embryogenesis.^18,20,21^ In the mutant cell, mitosis occurs normally but cytokinesis does not complete. The nascent two-cell embryo often reverts back to the single cell, and this “cytokinesis reversal” continues for multiple rounds. This results in multinucleated single cell embryos. It is possible that CS-containing matrix prevents refusion of newly formed cell membranes of the nascent two daughter cells, maintaining the two membranes separate.^25^ Also, CS may be required to fill the plasma membrane and eggshell, a chitin-based material.

In addition to vulva development and cytokinesis in embryos, CS regulates the migration of gonadal distal tip cells (DTCs).^26^ The unique migration paths of the DTCs during larval development determine the U-shape of the gonad arms (Figure 1). To elucidate the mechanisms of the directional migration of DTCs, a genetic screen was conducted to isolate mutations that affect migration patterns. The screen identified four mutations in two genes, *sqv*-*5* (ChSy) and *mig*-*22* (ChPF). These genes genetically interact with the components of the Netrin signaling system. Netrins are evolutionarily conserved secreted proteins that act as a chemoattractant (or a chemorepellent in some contexts) during axon guidance and cell migration. The genetic interactions between *sqv*-*5*/*mig*-*22* and the Netrin signaling components suggested that CS is required for Netrin-dependent guidance during DTC migration. CS was found to be localized in the BM surrounding DTCs. Although the mechanisms of CS requirement in Netrin signaling remain to be determined, it may modulate the receptor functions by creating extracellular milieu. It is also possible that CS sequesters the ligand or receptors to generate membrane microdomains of DTCs appropriate for signaling.

### *C. elegans* CS in longevity

In addition to the roles in morphogenesis, recent studies highlighted the impact of CS in longevity.^27,28^ *C. elegans* provides a powerful platform for drug screening: more than 100,000 compounds have been screened to date using this model, and 100 compounds were found to extend lifespan. Among these, CS has emerged as a promising compound.^27^ Oral uptake of CS increased *C. elegans* lifespan by 23–28%.^29^ Two mechanisms have been proposed for the effect of CS on longevity. First, it is known that CS supplementing delayed the progressive decline of collagen renewal and increased lifespan.^29^ A decrease in collagen biosynthesis and an increase in extracellular protease activity are general hallmark of aging across species.^30^ In *C. elegans*, prolonged expression of collagen is sufficient to extend the lifespan.^31^ These suggest that CS’s impact on aging is through ECM homeostasis. As a second possible mechanism, CS may slow aging by suppressing chronic age-related inflammation.^27^

Importantly, complementary to the feeding approaches, a recent genetic study demonstrated that endogenously elevated levels of CS indeed extend lifespan: a gain-of-function mutant of *mig*-*22* (ChPF) was found to live significantly longer.^28^ This mutant, *mig*-*22(gf)*, produces CS approximately twice as high as wild-type, which extends its lifespan by 30.6%. In addition to lifespan, *mig*-*22(gf)* also significantly increased healthspan assessed by body length, pumping rate, and mobility. These findings opened up future studies to investigate the molecular functions of endogenous and exogenous CS in aging. Since CS is a popular and widely used supplement, further studies should evaluate if it as a good candidate as a future geroprotective compound.

### *C. elegans* CSPG core-proteins

The early forward genetic screens discussed above-identified genes involved in the CS biosynthetic machinery but not CSPG core-protein genes. What core-proteins regulate CS-dependent biological processes, such as cytokinesis? Unlike HSPGs, CSPG core proteins are not well conserved between species.^32^ Therefore, the identification of CSPGs cannot rely on the sequence homology to mammalian counterparts. Olson et al. addressed these issues.^32^ Using a combination of partial purification of CSPGs by an anion-exchange column, chemical modification of the serine attachment residues, and mass spectrometry, nine CSPG core-proteins were discovered, which were named CPG-1 to −9 (Table 2).

Among them, two proteins, CPG-1 and −2, bear chitin binding domains, and they indeed bind to chitin.^32^ Both of them are expressed during embryonic development. RNAi knockdown of CPG-1 or −2 had no effect on embryonic viability, but simultaneous depletion of both resulted in the defect in embryonic cytokinesis, which mimics the *sqv*-*5* mutant phenotype. These observations suggested that CPG-1 and −2 redundantly function in cytokinesis of the first embryonic cell division. CPG-1 and −2 may fill the space between the chitin-based eggshell and the embryo to generate the formation of the hydrated extraembryonic space. Their simultaneous loss likely results in loss of this space, leading to the failure of stabilizing cytokinesis.

The attempt to identify new CSPGs was further expanded by a novel glycoproteomic approach.^33,54^ This method includes trypsin digestion of protein samples, enrichment of glycopeptides using strong anion exchange chromatography, and digestion by chondroitinase ABC. The chondroitinase treatment generates a glycopeptide bearing a residual hexasaccharide composed of the linkage region and a [GlcA-GalNAc] disaccharide. The glycopeptides are identified using nano-liquid chromatography-tandem mass spectrometry and evaluated by glycopeptide search algorithms. This protocol identified 15 novel *C. elegans* CSPGs on the top of the nine previously established core proteins, which added CPG-10 to CPG-24 to the list (Table 2). Some of them showed homology to vertebrate counterparts, including CPG-10/Col-XV/XVIII, CPG-16/Fibulin, and CPG-17/Papilin.

### *C. elegans* Papilin in development

The abovementioned glycoproteomic method identified Papilin as a CSPG (CPG-17).^33^ This gene had been known as *mig*-*6*, which regulates DTC migration.^37^ The *mig*-*6/Papilin* locus encodes two isoforms, long (MIG-6L) and short (MIG-6S) isoforms of Papilin, generated by alternative splicing. Both classes of alleles, *mig*-*6L* and *mig*-*6S*, show DTC migration defects but different phenotypes. Therefore, two protein products appear to regulate distinct aspects of DTC guidance. Genetic assays demonstrated that Papilin functionally interacts with collagen IV and ADAMTS metalloproteinases, suggesting that controlled degradation of the BM is a key factor to properly guide DTC migration path. As the study on *sqv*-*5* (ChSy) and *mig*-*22* (ChPF) described above indicated that CS is required for DTC migration,^26^ Papilin may be one core-protein which requires CS modifications to exert its function during DTC migration.

Papilin is also important for dendrite patterning.^38,55^ Ramirez-Suarez et al. studied the mechanisms of dendrite formation using multidendritic sensory neurons, the PVD neurons, as a model system.^38^ Experiments using laser and genetic ablation established that PVD dendrite patterning depends on axons of a neighboring neuron called the ALA neuron. Importantly, the study demonstrated that PVD dendrite control by ALA axons is contact-dependent rather than activity-dependent. It was proposed that the ALA axons generate an unknown guiding scaffold which is used by PVD dendrite extension. Mutations in Papilin and the Netrin pathway components were found to affect PVD dendrite patterning, probably through their functions in ALA axon guidance. It is worth noting that *sqv*-*5* and *mig*-*22* genetically interact with Netrin signaling during DTC migration as mentioned earlier.^26^ This study highlighted again a role of CSPG-containing BM in Netrin signaling control.

Papilin also appeared in a genetic screen for mutations that affect the shape of the pharynx.^39^ The pharynx is a feeding tube structure to pump the food to the gut, made of 80 cells surrounded by a thick BM (Figure 1). A class of mutant phenotype, *twisting of pharynx* (*twp*), is a useful model to identify molecules that are essential to maintain pharynx shape during growth. Interestingly, genetic screens using this model have yielded mutations in various ECM components, including Collagen, Laminin, Papilin, Perlecan, and Metalloproteinases. This suggests that maintaining the shape of this organ during growth may require appropriate adjustment of ECM stiffness as the organ enlarges during post-embryonic growth.

### Other *C. elegans* CSPG core-protein genes

There are a few other core-protein genes whose functions have been analyzed (Table 2). CPG-16/FBN-1 was found to be required for the epidermis to resist mechanical forces, preventing its deformation during embryogenesis.^36^ It is the *C. elegans* homolog of Fibrillins and is composed of multiple calcium-binding and non-calcium-binding EGF-like domains. CPG-16/FBN-1 is expressed in the embryonic epidermal cells and secreted to the apical surface. Therefore, it is likely to be a structural component of the embryonic sheath, the apical ECM of the embryonic epidermis. The embryonic sheath is a critical structure to protect the epidermis from excessive constriction and deformation caused by mechanical forces, namely a strong inward pulling force coming from the pharynx. The epidermis normally resists this, but upon impairment of *CPG*-*16/fbn*-*1* function, embryos cannot withstand mechanical forces, leading to severe deformation. Thus, CPG-16/FBN-1 provides a scaffold that protects the epidermis from deformation while it experiences mechanical forces during embryogenesis. Mutations in human fibrillin genes are associated with Marfan Syndrome (MFS), a connective tissue disorder mainly involving the cardiovascular, musculoskeletal, and ocular systems.^56^ The *C. elegans* model will contribute to understanding the underlying mechanisms of how a loss of Fibrillins causes mechanical collapse during MFS pathogenesis.

In addition to CPG-1 and −2, another ECM protein, HIM-4, was found to be required for normal cytokinesis, stabilizing the cleavage furrow.^34^ Depletion of HIM-4 resulted in cytokinesis failure, resulting in multinucleated cells in the germline. The glycoproteomic study described above revealed that HIM-4 is a CSPG (CPG-14).^33^ Although both classes of CSPGs, CPG-1/−2 and CPG-14/HIM-4, affect cytokinesis, their functions appear to be mechanistically distinct. CPG-14/HIM-4 is expressed in skeletal muscles and DTCs, and localized to the cleavage site in dividing germ cells where it promotes membrane ingression.^35^ Importantly, knockdown of Hemicentin-1, a vertebrate homolog of CPG-14/HIM-4, in mouse embryos results in cytokinesis failure,^34^ suggesting an evolutionarily conserved role of this class of molecules in cytokinesis.

### Sulfation of *C. elegans* CS

As mentioned earlier, *C. elegans* had been believed to synthesize only unsulfated chondroitin but lack CS. In 2016, however, using an enrichment protocol for sulfated GAGs and sensitized genotypes, a small amount of sulfated CS was detected in *C. elegans* (Table 1).^10,11^ A great abundance of unsulfated chondroitin might have masked the presence of the low levels of CS in the earlier studies. Analyses using reversed-phase ion-pairing HPLC and mass spectrometry detected sulfation at 4-*O*-position of galactosamine, and also at 6-*O*-position at a lower level.^10^ Importantly, in vitro and in vivo analyses determined that the gene C41C4.1 encodes a *C. elegans* CS 4-*O*-sulfotransferase (now named *chst*-*1*).^10,11^ The antibody CS-56, which recognizes sulfated CS but not non-sulfated chondroitin, strongly stained the cuticle, indicating that CS is mainly present in the cuticle of *C. elegans*. *chst*-*1* null mutants did not show any cytokinesis defects, suggesting that the function of CPG-1 and −2 does not require 4-*O* sulfation in the early embryonic cell division.^11^ This line of future studies will determine what *sqv*-dependent biological processes require CS sulfation, separating functions of CS versus chondroitin. Furthermore, a CS 6-*O*-sulfotransferase remains to be identified.

## DROSOPHILA CS

### *Drosophila* putative CSPG, Kon-tiki (Kon) in muscle development

Compared to *C. elegans*, the field of CS studies in *Drosophila* development is relatively young, and very few CSPG molecules have been identified to date: Kon-tiki (Kon), Multiplexin (Mp), and Windpipe (Wdp) (Table 2). Among these, Kon is a putative CSPG. It encodes the *Drosophila* homolog of mammalian NG2/CSPG4, but biochemical evidence for actual CS modifications of Kon has not been reported.

Kon was independently identified by two groups as a molecule essentially required for *Drosophila* embryonic muscle formation.^40,41^ Muscle development occurs through distinct steps- myoblast specification, migration, fusion, and attachment and establishment of a stable connection with tendon cells. Genetic screens to seek for molecules in muscle development identified *kon*, which encodes a single-pass transmembrane protein expressed on the muscle membranes.^40,41^ Its extracellular domain contains two globular-laminin modules, and the intracellular domain has a PDZ-binding motif. In *kon* mutant embryos, muscles fail to establish a stable connection with tendon. Consequently, mutant muscles detach upon contraction. Kon plays a similar role in the adult abdominal muscles^42^ and adult flight muscles.^43^

Kon’s function in the targeting and adhesion between embryonic muscles and tendons suggested that it functions as an ECM receptor/co-receptor. In fact, Kon has ability to recruit an integrin ligand, Thrombospondin, at the muscle-tendon junction.^44^ It was proposed that Kon mediates muscle-tendon adhesion by enhancing integrin signaling through localizing its ligand to the muscle membrane. Interestingly, ectopic expression of *kon* in tendon cells of *kon* mutant embryos partially rescues integrin ligand localization and the muscle detachment phenotype. This ability of Kon resembles the trans co-receptor activity of HSPGs where HSPG expressed in adjacent cells can mediate signaling *in trans*.^57–60^

### Kon in central nervous system repair

In mammals, NG2/CSPG4 is known to stimulate cell migration in vitro,^61^ and smooth muscle cells from NG2/CSPG4 mutant mice show reduced migratory activity.^62^ Therefore, Kon and NG2/CSPG4 may have an evolutionarily conserved functions in cell migration. In this context, a striking similarity in the functions of Kon and NG2/CSPG4 was observed during CNS repair.^45,63,64^ Upon CNS injury, the “glial regenerative response” can help regeneration and repair. Injury stimulates the proliferation of ensheathing glial cells, which can differentiate to remyelinate axons and partially restore functions. In mammals, NG2-positive glia are the repair cells that proliferate upon CNS damage, promoting functional recovery. In a *Drosophila* glial regenerative response model, *kon* expression is induced in glial cells by CNS injury.^45^ *kon* is required for glial proliferation, activation, and onset of glial differentiation during CNS injury repair. Thus, evolutionarily conserved NG2/CSPG4/Kon-dependent system regulates regenerative glial proliferation and differentiation in *Drosophila* and mammals.

### Mp in *Drosophila* development

Collagens XV and XVIII are the two members of a vertebrate collagen family termed multiplexins.^65^ Collagen XVIII bears HS chains, whereas collagen XV is modified with CS.^66^ *Drosophila* Mp is the only *Drosophila* homolog of Collagen XV/XVIII. Based on biochemical experiments using heparitinase or chondroitinase, Mp was reported as a CSPG.^46^

*Mp* was first identified as a modulator of motor axon pathfinding.^47^ *Mp* mutants are viable but show axonal pathfinding defects in the embryonic peripheral nervous system. More recent studies have shown that Mp regulates heart development.^48,49^ The *Drosophila* dorsal vessel is a single tube, which is divided into the non-contractile anterior aorta and the contractile heart domain. Mp is specifically expressed in the heart cardioblasts and is secreted into the luminal surfaces of the heart tube. In *Mp* mutants, the heart lumen is abnormally narrow, leading to impaired pumping. In contrast, overexpression of *Mp* in cardioblasts enlarges lumen size. Mp regulates the heart morphology and functions by enhancing Slit/Robo activity: Mp binds to Slit and increases Slit protein stability.

Mp also plays a role in blood cell homing.^50^ *Drosophila* blood cells, called hemocytes, reside in specific hematopoietic sites during development. In the larval stage, such hematopoietic sites, called the sessile hematopoietic pockets, are formed segmentally along the length of the body. Until recently, it was unknown how the sessile hemocytes attach to these specific sites. Csordas et al. found that the phagocytosis receptor Eater on the hemocyte surface physically interacts with Mp in the epidermal BM to anchor hemocytes to the body wall.^50^ Importantly, upon a clonal ectopic expression of *Mp* in the fat body, hemocytes were redirected to the surface of the clones, indicating that Mp is sufficient to anchor hemocytes.

### Wdp in *Drosophila* development

*windpipe* (*wdp*) encodes a single-pass transmembrane protein containing four leucine-rich repeats (LRR) in the extracellular domain. It was first identified as a molecule expressed at a high level in embryonic tracheal system.^67^ However, the function of this gene in tracheal development has not been reported to date. Instead, this gene was rediscovered to be a new component of the Jak/Stat pathway during adult gut homeostasis.^51^ Jak/Stat signaling is one of the key pathways that regulate the behaviors of intestinal stem cells (ISCs) during midgut homeostasis and regeneration. To identify novel downstream targets of the pathway, ChIP-Seq experiments were performed to search for the STAT92E-binding sites throughout the genome.^51^ The *wdp* locus was among the most promising candidates and it was confirmed that its expression is positively regulated by Jak/Stat signaling in the gut. Interestingly, Wdp was found to downregulate Jak/Stat signaling activity by promoting receptor internalization and degradation, thus forming a negative feedback loop. This feedback system is believed to play an important role in maintaining proper levels of Jak/Stat signaling activity during gut homeostasis and regeneration.

Further studies revealed additional information on this molecule. First, the above-mentioned glycoproteomic approach was applied to *Drosophila* samples and determined Wdp as a novel CSPG.^52^ Biochemical studies showed that Wdp is a major CSPG in *Drosophila*, which bears 4-*O* sulfated CS chains.^53^ Second, in addition to Jak/Stat signaling, Wdp negatively regulates three additional pathways: Hedgehog, Wingless (Wg), and BMP signaling.^52,53^ All of these four pathways negatively controlled by Wdp are morphogen signaling pathways positively regulated by HSPG glypicans.^68,69^ Given the structural similarities between CS and HS, it is not surprising that CSPGs have modulatory, supportive, and/or complementary functions to HSPGs. In fact, Wdp overexpression sequesters Wg ligand through its CS chains and reduces a pool of Wg ligand available to signal,^53^ consistent with the idea that HS and CS competitively function to fine-tune Wg signaling. Thus, Wdp is a general regulator of morphogen signaling pathways that are known as HS-dependent.

Despite strong impacts of *wdp* overexpression on the major developmental pathways, *wdp* null mutants are viable with relatively mild phenotypes. This appears to be due to the action of morphogen feedback systems. It is well known that morphogen pathways are controlled by multiple circuits of feedback regulation to buffer against genetic and environmental perturbations. Such multiple feedback loops are believed to contribute to the robustness of the morphogen systems.^70–73^ When a morphogen feedback system was perturbed by introducing a mutation in a key component of the loop, *wdp* mutation resulted in high levels of lethality and a variety of severe morphological defects.^53^ This suggested a possibility that Wdp is a novel player of the morphogen feedback buffering systems. Morphogen signaling can be an oncogenic pathway when dysregulated, and its precise dosage control is critical to prevent cancers. The functions of Wdp suggested that the “HS-CS dual PG co-receptor system” may provide an additional layer of dosage control to fine-tune signaling output.^52,53^

### CS in organ shape maintenance

As mentioned above, due to the minimal evolutionary conservation of CSPG core-proteins, the number of *Drosophila* CSPGs identified to date is limited. On the other hand, the *Drosophila* genome has homologs of CS biosynthetic enzymes, including chondroitin synthase (*Chsy*), chondroitin promoting factor (*Chpf*), chondroitin *N*-acetylgalactosaminyltransferase (*Csgalnact*), CS 4-*O* sulfotransferase (CG31743), and other orthologues of *C. elegans sqv* genes (Figure 2).^74–80^ To investigate the global function of CS in development, a null mutant for *Chsy*, the *Drosophila* homolog of human ChSy-1 and *C. elegans sqv*-*5*, was generated (Figure 2).^8^ Despite the complete lack of CS, a fraction of *Chsy* mutants survive to adult stage, which allows analyses of mutant adult organs. An assay using atomic force microscopy revealed an abnormally increased stiffness of the BM in the *Chsy* mutant ovary, showing the importance of CS in regulating BM mechanical properties.

The *Drosophila* ovary is highly active in contraction: it is surrounded by the muscle sheath, and its contraction contributes to the movement of the developing eggs posteriorly toward the oviduct. In *Chsy* mutants, the muscle sheath structures are impaired.^8^ In addition, the ovary contraction was significantly weakened in *Chsy* mutant ovaries: CS depletion impaired the amplitude but not the frequency of the ovary contraction. Thus, *Drosophila* CS is required for ovarian muscle integrity and contractile activity. As Kon is required for muscle-tendon attachment, this phenotype of *Chsy* appears to be consistent with a disrupted ECM-muscle linkage. Although it is unknown whether Kon plays a role in the ovary, a similar CSPG-dependent mechanism may function in the linkage of the ovarian muscle sheath.

**FIGURE 3:**
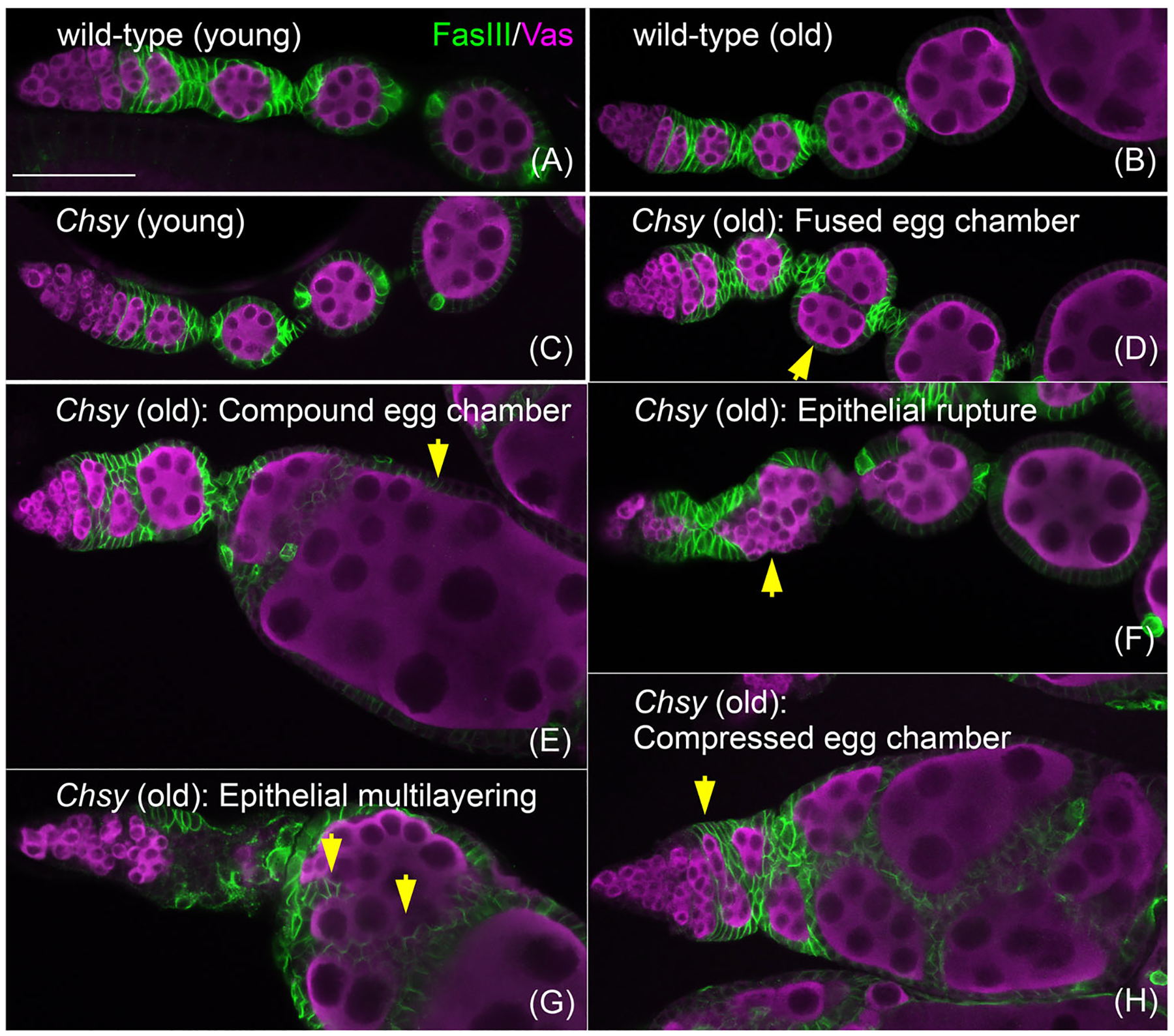
*Drosophila Chsy* mutants show a defect of organ shape maintenance. Ovarioles from wild-type (A and B) and *Chsy* mutant (C–H) females on day 3 (young; A and C) and day 21 (old; B and D) after eclosion. The ovarioles were stained for Fasciculin III (green, follicle cells) and Vasa (magenta, germ cells). Ovaries from old wild-type animals retained normal organ structures (B). Young *Chsy* mutant animals show no significant abnormality (C). The ovariole morphology from old mutants was massively altered (D–H). In these ovarioles, individual egg chambers showed abnormal shape and lacked a spatiotemporally ordered alignment of oogenesis. Five examples are shown for old *Chsy* mutants with different defects: fused egg chamber (D), compound egg chamber (E), epithelial rupture (F), epithelial multilayering (G), and compressed egg chamber (H) phenotypes (yellow arrows). Thus, *Chsy* mutants show a gradual decay of the gross organ structure in an age-dependent manner. Scale bar: 50 μm. Modified from Knudsen et al.^8^

The ovary is composed of 16–20 ovarioles made of progressively developing egg chambers. Oogenesis progresses with a proper organization of the growing egg chambers, each consisting of a germline cyst enclosed by a single layer of follicular epithelium (Figure 3A). Each egg chamber is separated by stalk cells. Morphological analyses of the *Chsy* mutant ovary showed that the initial assembly of the organ can occur relatively normally in the absence of CS (Figure 3C). However, *Chsy* mutants exhibit a gradual degradation of the gross organ architecture as they age^8^ (Figure 3D–H). Thus, CS is primarily required for organ shape maintenance, rather than morphogenesis, showing a striking contrast to HS’s functions in tissue patterning. The exact mechanisms for the age-dependent phenotypes of *Chsy* mutant ovaries are not yet completely understood. However, the early defects, the altered BM mechanical properties and a disrupted muscle anchorage, together with the impaired but continued contraction, may contribute to the progressive damage of the egg chamber organization.

### Sulfation of *Drosophila* CS

Compared to *C. elegans* CS, *Drosophila* CS contains a higher level of 4-*O*- sulfated disaccharide units (Table 1).^1,53^ The occurrence of 6-*O*-sulfation of galactosamine residue in *C. elegans* CS suggests that it may also exist in *Drosophila*,^10^ but this has not been demonstrated yet. Using anion exchange chromatography, *Drosophila* CSPGs can be separated into two groups with different charges: low- and high-affinity fractions to diethylaminoethyl cellulose.^53^ CS isolated from the high-affinity fraction contained 4-*O*- sulfated disaccharide but this was not detected in the low-affinity fraction. The observation suggested that two groups of CSPGs exist in *Drosophila*: one bearing 4-*O*-sulfated CS and another with non-sulfated chondroitin.

The only antibody shown to detect *Drosophila* CS to date is LY111.^8,53^ This antibody is known to recognize highly sulfated CS structures, such as 4-*O*-sulfated CS, GlcAβ1–3GalNAc(4S) or CS-A units.^81^ LY111 strongly stains the basement membrane in many *Drosophila* organs, including the larval wing disc and adult ovary.^8,53^ A novel gene, CG31743, encodes a protein orthologous to human CHST11. RNAi knockdown against CG31743 abolished the epitope of LY111, supporting the idea that CG31743 is a *Drosophila* CS 4-*O*-sulfotransferase.^53^

Comparison of basic features of vertebrate and invertebrate CS is shown in Table 1. In vertebrates, CS contains di-sulfated GlcAβ1–3GalNAc(4S,6S), or CS-E units. A sulfotransferase, *N*-acetylgalactosamine 4-sulfate 6-*O*-sulfotransferase (GalNAc4S-6ST) transfers sulfate to position 6 of 4-*O*- sulfated *N*-acetylgalactosamine in CS to yield CS-E units. A mouse strain lacking this enzyme fails to retain active proteases in the granules of bone marrow-derived mast cells^82^ and shows impaired osteoblast differentiation, resulting in low bone mass.^83^ In contrast, as 6-*O*- sulfation is scarce or undetectable in *C. elegans* and *Drosophila*, di-sulfated CS disaccharides have not been observed in these animals. Although Table 1 summarizes structural data only for CS, additional enzymes exist in vertebrates for epimerization of GlcA to IdoA to generate dermatan sulfate and for sulfation at the C-2 position of the IdoA. These enzymes are not identified in *C. elegans* and *Drosophila*.

## CONCLUDING REMARKS: DEVELOPMENTAL GENETICS MEETS GLYCOBIOLOGY

Functional studies of CS using the genetically tractable model organisms, *C. elegans* and *Drosophila*, with a combination of unbiased genetic screens (forward genetics) and targeted research of CS-related genes (reverse genetics), revealed its various roles during development. Only a limited number of evolutionarily conserved CSPG core-proteins have been identified in these species. Developmental roles of these molecules, including Papilin, Kon/NG2, and Mp/Collagen XV/XVIII, showed both conserved functions and unique actions specific to each species. In contrast, the CS biosynthetic mechanisms are largely conserved from these species to humans. This will allow future functional analyses of human patient variants of the biosynthetic enzymes to elucidate pathophysiological mechanisms and etiology of human disorders. Importantly, disease models in *C. elegans* and *Drosophila* are known to be highly useful for in vivo drug screening to identify novel inhibitors for therapeutic purposes.^84–86^

CS appears to have functions both similar to and distinct from HS. For example, *Drosophila* Wdp modulates HS-dependent morphogen pathways.^51–53^ On the other hand, analyses of *Chsy* mutants, the first *Drosophila* CS-deficient model, showed roles of CSPGs as a regulator of tissue mechanical properties.^8^ CS is critical in organ shape maintenance during aging rather than pattern formation, unlike essential roles of HS in tissue patterning.

Interestingly, compared to vertebrate CS studies, which highlighted the importance of sulfated domains, studies in *C. elegans* and *Drosophila* suggested significant roles of unsulfated chondroitin. Future analyses of CS sulfotransferase mutants and the comparison of their phenotypes with CS polymerase mutants will define distinct roles of sulfated CS and unsulfated chondroitin. Furthermore, CS/HS structural analyses in *Drosophila* have been performed using the whole-body samples (third instar larvae and adult flies) to date (Table 1). Tissue- and cell type-specific structural profiles of GAGs remain to be elucidated.

## Figures and Tables

**TABLE 1:** Disaccharide compositions of vertebrate and invertebrate CS.

	ΔDi-0S (%)	ΔDi-4S (%)	ΔDi-6S (%)	ΔDi-diSE (4S,6S) (%)	ΔDi-diSD (2S,6S) (%)	Total amount	References
Human urinary bikunin	64.5	35.5	ND	ND	ND	-	[1]
Human plasma	63.4	36.6	ND	ND	ND	13.1 (μg/ml)	[2]
Human serum	31.4	67.0	1.6	ND	ND	24.7 (μM)	[3]
Human urine	8.0	33.0	56.6	2.4	ND	6.6 (nmol/mg of creatinine)	[4]
Mouse cerebral cortex	6.4	89.5	2.2	1.2	0.7	-	[5]
Mouse hippocampus	4.6	89.0	4.6	1.0	0.7	-	[5]
Shark cartilage	1.7	15.4	73.0	0.6	9.3	-	[6]
*C. elegans*	100.0	ND	ND	ND	ND	3070 ng/mg dry tissue	[1, 7]
*Drosophila* adult	69.1	30.9	ND	ND	ND	116.6 ng/mg dry tissue	[8]

*Note*: CS disaccharide compositions are compared between vertebrate and invertebrate species. CS from mammals contains a higher percentage of ΔDi-4S mono-sulfated unit than ΔDi-6S, while CS from sharks shows the opposite pattern. ΔDi-0S, HexA-GalNAc; ΔDi-4S, HexA-GalNAc(4S); ΔDi-6S, HexA-GalNAc(6S); ΔDi-diSE, HexA-GalNAc(4S,6S); ΔDi-diSD, HexA(2S)-GalNAc(6S); n.d., not detected.

**TABLE 2:** *C. elegans* and *Drosophila* CSPG core-protein genes.

Gene	Domain structures	Vertebrate homologs	Functions	Reference
*C. elegans*				
CPG-1/cej-1	Chitin binding Peritrophin-A domain	-	Cytokinesis	[32, 33]
CPG-2	Chitin binding Peritrophin-A domain	-	Cytokinesis	[32, 33]
CPG-3	N-terminal low-complexity domain	-	-	[32, 33]
CPG-4	C-terminal low-complexity domain	-	-	[32, 33]
CPG-5/clec-87	C-type lectin domain	CLEC2L, KLRB1, KLRG1	-	[32, 33]
CPG-6/clec-88	C-type lectin domain	KLRF2	-	[32, 33]
CPG-7	Disordered and low complexity domains	-	-	[32, 33]
CPG-8	Disordered and low complexity domains	-	-	[32, 33]
CPG-9	Disordered and low complexity domains	-	-	[32, 33]
CPG-10/CLE-1A	Fibronectin type-III domain, Collagen domain, Endostatin domain	Collagen XV/XVIII	-	[33]
CPG-11/col-164	Transmembrane domain, Cuticle collagen domain, Collagen domain	Collagen VI	-	[33]
CPG-12/clec-180	C-type lectin domain, Coiled coil domain	C-type lectin domain-containing protein 180, Zonadhesin (M. *musculus*)	-	[33]
CPG-13/dur-135/dur-1	Coiled coil domain	-	-	[33]
CPG-14/him-4	Immunoglobulin domain, Immunoglobulin I-set domain, Calcium-binding EGF domain	Hemicentin	Cytokinesis	[33–35]
CPG-15/lpr-5	-	-	-	[33]
CPG-16/fbn-1/let-821	Calcium-binding EGF domain, EGF domain	Fibrillin	Protection from mechanical forces	[33, 36]
CPG-17/Mig-6/Papilin	Thrombospondin type 1 domain, ADAM spacer 1 domain, Kunitz-type serine proteinase inhibitor domain and PLAC domain	Papilin	DTC migration, ALA axon guidance, Pharynx shape maintenance	[33, 37–39]
CPG-18	Disordered domains	-	-	[33]
CPG-19	Disordered and low complexity domains	-	-	[33]
CPG-20	Reprolysin family propeptide	-	-	[33]
CPG-21	Somatomedin B domain	-	-	[33]
CPG-22	Dopamine β-monooxygenase (DOMON) domain	-	-	[33]
CPG-23	Disordered and low complexity domains	-	-	[33]
CPG-24	Disordered and low complexity domains	-	-	[33]
*Drosophila*				
Kon-tiki (Kon)	Laminin G-like domain	NG2/CSPG4	Muscle development, CNS repair	[40–45]
Multiplexin (Mp)	N-terminal thrombospondin-related domain, Interrupted triple helix, C-terminal endostatin domain	Collagen XV/XVIII	Motor axon pathfinding, Heart development, Blood cell homing	[46–50]
Windpipe (Wdp)	Leucine-rich repeat domain		Negative regulation of Jak/Stat, Hh, BMP, and Wg signaling	[51–53]

## Data Availability

All data are contained within the manuscript.

## Abbreviations:

BM: basement membrane
ChGn: human chondroitin *N*-acetylgalactosaminyltransferase
ChPF: human chondroitin promoting factor
Chpf: *Drosophila* chondroitin promoting factor
ChSy: human chondroitin synthase
Chsy: *Drosophila* chondroitin synthase
CNS: central nervous system
CPG: chondroitin proteoglycan
CS: chondroitin sulfate
Csgalnact: chondroitin *N*-acetylgalactosaminyltransferase
CSPG: chondroitin sulfate proteoglycan
DTC: distal tip cell
ECM: extracellular matrix
ER: endoplasmic reticulum
frc: fringe connection
GAG: glycosaminoglycan
Gal: galactose
GalNAc: *N*-acetylgalactosamine
GalNAcT: *N*-acetylgalactosaminyltransferase
GalT: galactosyltransferase
gf: gain-of-function
GlcA: glucuronic acid
GlcAT: glucuronosyltransferase
Glu: glucose
HS: heparan sulfate
HSPG: heparan sulfate proteoglycan
ISC: intestinal stem cell
Kon: Kon-tiki
LRR: leucine-rich repeats
MFS: Marfan Syndrome
Mp: Multiplexin
oxt: peptide O-xylosyltransferase
Ser: serine
sgl: sugarless
sqv: squashed vulva
twp: twisting of pharynx
UDP: uridine diphosphate
Uxs: UDP-xylose synthase
Wdp: Windpipe
Wg: Wingless
Xyl: xylose
